# The phenomenon of co-morbid physical and mental illness in acute medical care: the lived experience of Australian health professionals

**DOI:** 10.1186/s13104-015-1264-z

**Published:** 2015-07-07

**Authors:** Jo-Ann Giandinoto, Karen-leigh Edward

**Affiliations:** Faculty of Health Sciences, School of Nursing, Midwifery and Paramedicine, Nursing Research Unit, Australian Catholic University/St Vincent’s Private Hospital Melbourne, Locked Bag 4115, Fitzroy, MDC 3065 Australia

**Keywords:** Phenomenology, Physical health, Mental health, Co-morbid, Australian health professional, Nursing, Medical care setting

## Abstract

**Background:**

An estimated 30–50% of patients admitted to acute medical care settings experience co-morbid physical and mental illness. Research suggests that health professionals in these settings find managing this patient group challenging. A number of studies have investigated health professional’s attitudes and perceptions however there is limited research that investigates the lived experience in a current Australian healthcare context. The aim of this study was to explicate an in-depth description of the health professional’s experience when caring for patients experiencing co-morbid physical and mental illness in Australian acute medical care settings.

**Methods:**

A phenomenological design was undertaken with six participants representing nursing and medical disciplines. In 2013–2014 one-on-one semi-structured interviews were used and the data collected underwent thematic analysis using an extended version of Colaizzi’s phenomenological inquiry.

**Results:**

Six themes emerged including—challenging behaviours, environmental and organisational factors, lack of skills, knowledge and experience, hyper-vigilance and anxiety, duty of care and negative attitudes with an overarching theme of *fear of the unknown*.

**Conclusions:**

Staff in acute medical care settings were unsure of patients with mental illness and described them as unpredictable, identifying that they lacked requisite mental health literacy. Regular training is advocated.

**Electronic supplementary material:**

The online version of this article (doi:10.1186/s13104-015-1264-z) contains supplementary material, which is available to authorized users.

## Background

Disproportionate rates at which people diagnosed with serious mental illness (SMI) (such as; psychotic and schizophrenia type disorders and depression) who additionally experience co-morbid physical health conditions has gained increased recognition [1]. Mental illness itself is one of the top ten disabling illnesses worldwide, making up 13% of the total global burden of disease [2]. In Australia between one and five people will experience a mental illness in a 12 month period [3]. The World Health Organisation (WHO) [2] reports that people diagnosed with SMI have a 40–60% increased chance of dying prematurely when compared to people who do not experience mental illness and it is estimated that they have a reduced lifespan of between 10 and 30 years [4]. People experiencing mental illness have an increased risk of developing chronic physical illnesses such as cardiovascular disease, diabetes, cancer and HIV/AIDS. Mental illness and its symptomatology increases the risks of physical illness because it can lead to; poor management of illness, financial disadvantage, stigma, medications and lifestyle factors such as inactivity and substance misusing behaviours [5]. The interactional nature of physical and mental illness is apparent, for example as mental illness interacts with a person’s physical health, one’s physical condition may negatively affect a person’s mental state [6]. An example of this occurring can be drawn from a situation where a person who experienced an acute myocardial infarction may contribute to the onset of depression.

With the introduction and implementation of the National Mental Health Policy 1992, mental healthcare services in Australia underwent a reformation with lasting effects that are still relevant today. Previously, mental health services were custodial and segregated from mainstream healthcare. This policy, including a joint statement from Health Ministers of Australia, acknowledged that a reform of mental healthcare from institution to a community oriented approach was needed [7]. The mainstreaming of mental health services occurred in the hope that people experiencing mental illness would be exposed to less discrimination and stigma and improved equity of access to healthcare and other linked services [8]. The de-institutionalisation of mental healthcare meant that there was a significant reduction in mental health inpatient beds and a rise of acute mental healthcare occurring in non-mental healthcare acute hospitals [9]. Subsequently the healthcare needs of people experiencing a mental illness present for services in larger volumes in general practice, acute medical hospitals, rehabilitation centres and in ambulatory care [10].

An estimated 30–50% of patients admitted to acute medical care settings experience co-morbid physical and mental illness [8]. Health professionals, including nurses and medical staff, in the acute medical care setting such as emergency departments, medical-surgical wards, intensive care units and general-medical wards are therefore in regular contact with patients who experience mental illness as a co-morbidity to a physical condition. Some literature suggests that health professionals in these “non-mental health” settings find the complex care of these patients challenging and consequently the patient’s experience of care can be poor [11, 12].

Some research reveals fear prevents health professionals from effectively caring for people who experience mental illness in the acute medical care setting [13, 14]. The findings included expressed concerns of fear towards patients with a mental illness due to an assessment of risk and dangerousness causing healthcare staff to be concerned for their own safety and that of other patients [15]. Brinn [16] also discusses negative emotions expressed by nurses who cared for those with co-morbid mental illness, such as fear and wariness owing to an expectation of aggression. MacNeela, Scott, Treacy, Hyde and O’Mahony [17] found that most nurses adopted a “risk attitude” when looking after patients experiencing mental illness. They explained that the stereotyped perceptions of patients experiencing a mental illness such as non-compliance, absconding and a violence risk, altered their experience and expectations of care and moreover this justified the need for chemical and or physical restraining of patients. Stigma towards people with mental illness can negatively affect the relationship between the patient and the health professional. Despite having medical knowledge, some health professionals still hold stereotyped views about mental illness paralleled to those found in the general public [18]. This can create real barriers to providing care [15, 19]. For nurses, undergraduate training is often not considered adequate for developing mental health literacy (i.e. diagnosis, psychopharmacology, management, legislation) [20–22]. In addition, the physical environment offers a number of barriers to providing comprehensive and timely care for people experiencing mental illness in the acute medical setting context of a hospital. Patients experiencing mental disorders often require a therapeutic environment to aid recovery and enable communication with staff. When patients are considered as not fitting into the purpose of the environment, health professionals’ attitudes can alter; frustrations increase and fears become apparent with stigmatizing and stereotyping behaviours presenting [12, 23].

A recent systematic review revealed a paucity of research using a phenomenological framework and its methods to examine this phenomenon in the Australian context existed [15]. It is therefore timely that research investigating the phenomenon of health professionals caring for patients with a co-morbid mental and physical illness in acute medical care settings be undertaken using a phenomenological method. This study aimed to explicate an in-depth description of the phenomenon of health professional’s experiences whilst working with patients experiencing co-morbid mental illness in acute medical care settings and to understand health professional’s attitudes and perceptions towards people experiencing mental illness in these settings. The findings will provide a current perspective of health professional’s experiences in an Australian context in regards to caring for people with physical and mental health co-morbidity in non-mental health settings. The findings also have the potential to inform professional development strategies with the inclusion of such information in teaching modules and clinical education to assist staff working in acute non-mental health medical care environments.

## Methods

### Research design

This study used a phenomenological approach to obtain an in-depth description of the phenomenon of health professionals’ experiences of caring for patients experiencing co-morbid mental and physical illness in non-mental health acute medical care settings. Phenomenology has been adopted by many nurse scholars as a philosophical perspective, a mode to practice and as a research method to develop deep holistic descriptions and understandings of common, day-to-day activities [24–26]. The goal of understanding the lived experience in phenomenology is harmonious with the principles of nursing where the holistic understanding of the individual forms the basis of the nurse–patient relationship [27] and it is the goal of this research to obtain an in-depth description of health professional’s experiences. Unlike phenomena existing in the natural world that can be measured or quantified, phenomena of lived experiences may change over time and social contexts—thus a rigorous qualitative method should be used for its inquiry.

The phenomenological methodology for this study was based on an adaptation of Colaizzi’s [28] phenomenological mode of enquiry. According to Colaizzi, the fundamental structure of the phenomenon under investigation is the articulation of the moment in the researcher’s mind and understanding is based on accepted presuppositions and situational circumstances. In this context, Colaizzi’s phenomenological approach of inquiry can provide knowledge about aspects of a person’s lived world that cannot be accessed through observation alone.

In 2007, two Australian qualitative researchers, Edward and Welch, extended Colaizzi’s empirical existential approach of phenomenology by exploring phenomena via the interpretation of a symbolic representation of the experience provided by the participants [29]. The researchers posit that for participants using words alone to describe the phenomenon in question may not suffice to capture the essence of the participant’s experience and hence proposed the use of a symbolic representation (i.e. the use of a metaphor, poetry, painting, music, artefact, song or any image in the mind of the participant) may assist participants to communicate their experience [29]. The additional step allowed the researchers to gain deeper access and further explore the phenomenon, allowing a more holistic description of participant’s lived world. The use of symbolic representation of a human experience is a powerful vehicle to bring forth the complex inner world that makes up the experience so it can be understood and shared with others [29]. Analysing the symbolic representation of the experience increased the understanding of both what was spoken and unspoken in their participant’s narratives [29]. They concluded that the use of symbolic representation brought a new dimension to the participant’s experience in which they had not previously articulated, this in turn facilitated the understanding to individual’s intended meanings and led to a greater understanding of the experience which may not be necessarily understood if verbal language alone was used [29]. The use of symbols and the researcher’s interpretation of the symbolic representation put forward by the participants are especially valuable in adding to the rigour, in particular confirmability of the research data.

### Participants

A purposive sampling method was used to recruit participants from a number of metropolitan hospitals in Melbourne, Australia. Health professionals were invited, by placement of a research flyer in public places (e.g. gymnasiums and shopping centres) which called for potential participants to contact the researcher if they have had the experience of caring for patients with co-morbid physical and mental illness whilst working in a non-mental health acute medical care setting. This purposive sample was selected from those responding to the advertisement as well as subsequent snowball sampling [30] until data saturation was achieved. Data saturation occurred when repeated themes from participants arose and no new evidence emerged. The participants included in the study met the following inclusion criteria—any health professional working in non-mental health acute medical care settings who had cared for patients with co-morbid physical and mental illness, male or female health professionals and were between the ages of 18–65 years as this is general representation of health professionals in the hospital workforce. This age range also provided a varying range of experience, skill level and tertiary qualification. The participants who were excluded from the study were mental health professionals and those residing outside of metropolitan Melbourne, Australia, due to feasibility of travelling and time constraints.

### Data collection

Data were collected between October 2013 and April 2014 using one-on-one semi-structured interviews with one of the researchers. The interviews lasting approximately 30–60 min in duration were digitally recorded and later transcribed verbatim. The aim of the interview was to obtain descriptions of the life world view of the participants so that the researchers interpreted and made meaning of the phenomena described [31]. The researcher and participant met at a mutually convenient location and the researcher facilitated the interview allowing participants to speak freely and openly, encouraging them to provide a rich description of their experiences. The research questions were open-ended and this ensured the researcher did not include any of their prejudices or influence the discussion in any way.

The focus question for this study which guided the semi-structured interviews was:

“Can you describe your experience when working in an acute medical care setting and caring for a patient with a co-morbid mental and physical illness?”

Any subsequent questions during interviews were recursive in nature and examples of these were as follows:

“Can you please describe any strategies you may have used to provide care to these patients with co-morbid physical and mental illness?”

“Can you describe any barriers to care in the acute care setting that you encountered?”

As the study also involved the interpretation of symbolic representations, questions such as this was asked at the close of the interview:

“When you think of the experience of caring for a patient with a co-morbid physical and mental illness on the acute medical ward, what image do you have in your mind?” Or “What kind of symbolism would you use to represent your thoughts about caring for this patient in your care on the acute medical ward?”

### Ethical considerations

The project involved the use of human participants, and an ethics application was approved by the University’s Human Research Ethics Committee. The study received the protocol number 2013 215V. Issues relating to consent, confidentiality, anonymity, storage of information and risk contingencies were addressed through the process of obtaining ethical approval.

### Data analysis

Data analysis was guided by Edward and Welch’s [29] extended version of Colaizzi’s phenomenological approach. The extended version outlined by Edward and Welch [29] has eight steps of data analysis, with the additional step of analysis of the symbolic representations presented by the participants at the interview. This method of data analysis included the following steps undertaken by the researchers (also represented in Additional file1: Table S1).

Following transcription of the interviews, transcripts (narrative data) were read (and re-read) to gain a general sense of the key themes that occurred in each of them (step one). The transcripts were then highlighted for key phrases (significant statements) which related to the health professionals’ experiences of caring for a patient experiencing co-morbid mental and physical conditions (step two). These significant statements were extracted and numbered, then were analysed with reference to the original transcript to formulate meanings (referring the statements back to the interview ensured that they were interpreted in the context described by the participant) (step three). Each formulated meaning was then clustered into themes (group of statements that hold similar meanings) (step four). The themes were then integrated to form an exhaustive description of this experience which takes into account all of the formulated meanings given to each of the participant’s significant statements (step five). In step six the researcher interpreted the symbolic representations provided by the participants, this is the additional step added by Edward and Welch [29]. The method then allows for the development of two fundamental structures one from the narrative data and the other from the researcher’s interpretation of the symbolic representation. The development of the two fundamental structures allowed for triangulation to occur, ensuring that the true essence of the phenomenon as experienced by the participants was described. The transcripts were returned to participants for verification at the beginning of the analysis, where they were invited to add or change any of the transcript data.

### Rigour

The rigour of the research was tested upon the four criteria developed by Lincoln and Guba [32] being credibility, transferability, dependability and confirmability. These criteria are considered the ‘gold standard’ which to judge the trustworthiness of qualitative research [33]. To ensure credibility and dependability member checking was used where transcripts were returned to the participants to ensure accuracy and also provided an opportunity for participants to clarify or add to their transcript. Peer debriefing between researchers occurred to address any issues related to prejudice, inaccurate interpretations and consensus on emergent themes. The fundamental structure developed from the narrative data was confirmed against a second fundamental structure which the researchers developed from the researcher’s interpretation of the participant’s description of their symbolic representations. This further ensured a synchronicity between the researcher’s interpretations and the explanations made by the participants of their experience. A clear research audit trail, interview notes and reflexive journal were also maintained throughout the study.

## Results

### Participant context

There were six participants involved in this study. The participants were three females and three males, with four being registered nurses and two medical doctors, of which one was in his final year of medical study. The age range of the participants was from 21 to 37 years. They had all had worked on or were currently working in non-mental health acute medical care settings of which included the emergency department, medical-surgical wards, general-medical wards, medical specialty wards such as neurosciences and urology and peri-operative areas.

### Emergent themes

Emergent themes were explicated from the participant’s transcriptions and these were: managing challenging behaviours; environmental and organisational factors; lack of skills, knowledge and experience; hyper-vigilance and anxiety; duty of care; and negative attitudes.

#### First emergent theme: managing challenging behaviours

Managing challenging behaviours was identified by participants in this study as an area for consideration when working with patients experiencing a mental illness in an acute medical care setting. The challenging behaviours described by the participants included patients who were disruptive, demanding, difficult, non-compliant, aggressive and agitated, unpredictable and dangerous. Patients experiencing a co-morbid mental illness may display unusual behaviours that disrupt the routine of busy wards such as medical-surgical wards and disturbed the comfort of fellow patients in wards where patients shared rooms. This is described by one participant stating:

“The patient… was arching up a bit and being quite disruptive… didn’t make it

easy for the three other patients in the room… this person in the bed next to

them was always smoking, swearing and carrying on…” (Participant 1)

#### Second emergent theme: environmental and organisational factors

The participants described a number of barriers within the physical care environment and some organisational factors that impeded the care of patients with a co-morbid mental and physical illness. The environment of acute medical care setting was described as unsafe and inappropriate to effectively manage the care of patients with co-morbid mental and physical illness. This opinion often coincided with the description that patients experiencing a mental illness were unpredictable and may become violent with the environment offering little protection to patients and staff alike. One participant remarked:

“…you know this patient can lunge at you and stab you… and they are

completely unpredictable… in the emergency department it was dangerous

because he picked up a piece of equipment and the cops were there so

they had to pepper spray him!” (Participant 6)

#### Third emergent theme: lack of skills, knowledge and experience

In addition to factors external to healthcare staff, participants reported times when they felt unprepared to adequately care for a patient experiencing mental illness. One participant stated:

“Truthfully I haven’t done a mental health assessment since I was a student.

I wouldn’t know where to start…” (Participant 2)

Another participant stated:

“It was really the unknown… I had never cared for anyone in alcohol

withdrawal and I just knew from that they could be physically and verbally

aggressive but didn’t really know how to deal with it, I wasn’t adept with

what would I do, I didn’t know how to act.” (Participant 4)

#### Fourth emergent theme: hyper-vigilance and anxiety

Hyper-vigilance and fear when working with patients with a mental illness in the acute medical care setting were highlighted by participants in this research. Hyper-vigilance related to safety of self and others. As one participant stated:

“My past experiences or when I hear about other staff’s past stories

of when they have cared, for example, negative stories about IV drug

users, I always suspect the worst and am always hoping it is going to

be better than what I am thinking…” (Participant 2)

Another participant further described their experience as:

“There is this struggle with dealing with an aggressive patient on a

surgical ward we don’t really know what to do and no one has a

set role in a crisis. It can be scary…Especially when a patient has me

watching them 24-7 it can make my other patients feel uneasy because

they don’t know why this patient has to be watched all the time, they need

extra reassurance.” (Participant 3)

#### Fifth emergent theme: duty of care

The participants for the majority identified a professional responsibility to effectively care for patients experiencing co-morbid physical and mental illness. However, they identified and acknowledged the difficulties and limitations in their knowledge and environment the health professionals were often concerned with patient’s safety and were vigilant in ensuring patients received adequate care. Participant three described the following experience:

“The patient had a background of IV drug use and a schizoaffective

disorder… I felt like I didn’t do my job properly. This patient really

wasn’t in a good position to make a good judgement call about his

health and when he left the ward I kind of felt like I had let him down.”

(Participant 3)

#### Sixth emergent theme: negative attitudes

Caring for patients with a co-morbid physical and mental illness in acute medical care settings evoked negative emotions in the majority of participants. All of the participants recalled events with patients that were negative in nature. A few of the participants acknowledged that when thinking of mental illness it is the most violent, “crazy” and offensive patient they recall.

One participant described health professional’s attitudes about a patient with a co-morbid mental and physical illness diagnosis:

“It was sort of surprising because as soon as that psychiatric label

was mentioned it changed the ball game… the phrase that “psych is

involved” it seemed to change attitudes. Healthcare staff might have the

idea that all “psych” patients are crazy. I felt that it is impossible to

talk to them normally because their ideas are completely different from mine

and all that goes with being crazy.” (Participant 5)

Some participants, however, based on their past experiences and professional knowledge did attest to holding more positive attitudes towards patients experiencing mental illness when they compared themselves to some of their colleagues who may not have had the same experiences or affinity to caring for people experiencing mental illness. Participant three described their personal interest in mental health care:

“I have an interest in mental health but I have found that other staff on the

surgical ward who have been there for a while… often say I hate specialling these patients…

staff do judge patients based on past experiences.” (Participant 3)

Participant six described how their experience of working in a mental health ward on a clinical rotation shaped their attitudes and understanding of mental illness and considers this when caring for patients in the acute medical setting:

“From my experience I am not afraid of mental illness anymore and I feel

like I can actually help someone with a mental illness… I find that other staff

who have not done psychiatric [rotation] don’t actually have that experience

and feel threatened or are actually afraid to treat these patients.” (Participant 6)

### Analysis of symbolic representation

The modification of Colaizzi’s phenomenological method for data collection and analysis by Edward and Welch allowed for participants to provide a symbolic representation of their experiences of caring for patients with co-morbid mental and physical illness. The symbolic representation and researcher’s interpretation is presented in Additional file 2: Table S2.

### Overarching emergent concept

Based on the study’s findings an overarching emergent concept that was developed to define the phenomenon of caring for a patient with co-morbid physical and mental illness in an acute medical care setting is a *fear of the unknown*. Health professionals are often unprepared and lack the requisite knowledge to appropriately manage patients. There exists a perception that patients experiencing mental illness can be difficult, challenging, violent, unpredictable and aggressive and this causes tension in health professionals when they are confronted with patients with a label of mental illness in an environment that is not necessarily equipped to support effective care delivery.

## Discussion

The findings of this study were not unlike the findings of Atkin, Holmes and Martin [14] and Björkman, Angelman and Jönsson [18] who revealed that nurses often identified and experienced difficulty in managing unusual behaviours their patients displayed. Hopkins [34] in an ethnographic study found that nurses on medical wards described patients with complex needs as those who blocked the flow of routine on a busy ward causing nurses to feel challenged and resentful, especially if they are unprepared to manage such behaviours. Similar to earlier research the findings of this study also suggest health professionals had pre-determined ideas that a patient with a mental health diagnosis would be a disruption to daily routines suggesting negative stereotypes for patients experiencing a mental illness [16, 35, 36].

Barriers such as environmental and organisational barriers were also highlighted in this study. Plant and White [37] discuss these barriers in the environment in their research. These included; lack of support, time, resources and adequate privacy to treat patients with dignity. In their sample the frustration and helplessness experienced by nurses in the emergency department led nurses to professional burn-out. Patients were referred to as not fitting into the environment of the acute medical care setting as they were described as being intrusive, non-compliant in addition to insufficient resources on the ward to assist in the effective management of these patients. Similarly, Zolnierek and Clingerman [23] found that the experience of caring for a patient with a co-morbid mental illness on a medical-surgical ward was difficult as the patient was considered a ‘misfit’ in the environment by disrupting routines and other patients. In addition, the environment in the acute medical care setting has been described as exacerbating symptoms of mental illness due to routine physical care, lack of space and privacy and noise were considered unsuitable for the care of patients experiencing mental illness [36]. Other factors in the environment identified in the literature supports the findings of this study that health professionals being challenged by time constraints to talk to patients about their mental illness and lacked requisite mental health care support and resources [38].

When healthcare staff feel like they are working outside their scope or expertise they can become frustrated and disempowered resulting in negative consequences for both the patient and the health professional [17, 37]. Potential moderators for improving attitudes towards mental illness include improving knowledge, self-awareness and increasing exposure to people with a mental illness which was also identified by some of the participants of this study. Priami, Plati and Mantas [39] and Arvaniti, Samakouri, Kalamara, Bochtsou, Bikos and Livaditis [40] found too that once nurses received education and support by the way of mental health liaison staff including a sense of familiarity regarding the care of patients experiencing mental illness their attitudes became more positive compared to baseline measures. On the other hand education and increasing exposure does not always predict more positive attitudes as described by a few of the participants [16], misrepresentation of particularly negative experiences with patients or hearing about only the “worst” cases of patients experiencing mental illness can often bias opinions towards the negative.

Fear and hyper-vigilance from staff caring for those with dual diagnosis in non-mental health settings has also been reported in other research [41]. These patients often elicit stigmatizing behaviours or attitudes potentially impacting care [35]. Nurses report a lack of understanding, skills and expertise to manage patients who can be unpredictable leading to high levels of uncertainty and tension [23]. Sharrock and Happell [8] identified where nurses had expressed attitudes such as doing the “right thing” by their patients but acknowledge that the challenges such as inadequacies in undergraduate educational preparation and the barriers in the acute medical environment as also impacting. Health professionals can strive to do their very best but unfortunately a label of mental illness can inadvertently impact the care delivery offered to such patients [42]. The bias towards negative emotions and attitudes is not dissimilar to the misunderstandings and stigmatising attitudes that are found in attitudes towards mental illness in people of the general population [18]. Increased levels of familiarity, education and exposure are thought to protect against healthcare staff, [43, 44] however the positive effects of education can decay over time especially where health professionals are exposed to mostly negative experiences.

## Limitations

This study has some limitations, the use of a phenomenological design for methodology it is inherent that the data is not considered generalisable. The intent of this research was not to generalise but to acquire and in-depth description of the phenomenon of caring for a patient who is experiencing a co-morbid physical and mental illness via the perspective of Australian health professionals which may be transferred to other similar healthcare settings. Where interviewing as a method for data collection can provide a compelling and rich source of information, participants may have found it difficult to articulate experiences where they may have delivered substandard care and this may have influenced their responses. Member checking and discussing issues related to anonymity and de-identification of data including keeping a research audit trail assisted to maintain credibility of these research findings.

## Conclusions

The findings of this research investigating the phenomenon of caring for patients experiencing co-morbid mental and physical illnesses in acute medical care settings via the perspective of the health professional echoed the findings in the previous research. The experience of caring for patients with this complex co-morbidity in acute medical care poses challenges for the generalist health professional. In particular, factors intrinsic to the health professional including knowledge, skills, attitudes and values towards mental illness; factors related to the patient including challenging behaviours and labels of mental illness and factors related to the hospital environment and professional issues such as scope of practice, safety risks, lack of time, hospital routines and resources. The overarching emergent concept identified by this study was a fear of the unknown, where staff were unsure and patient behaviours were essentially described as unpredictable. Recommendations based on the findings of this study include the provision of environmental and organisational supports for generalist healthcare staff by provision of mental health liaison consultation staff. A further recommendation is that all health professionals working with patients should be subject to annual mandatory competency training in basic mental health care to increase confidence, self-awareness and enhance clinical skills and expertise. At present this level of attention to mental health literacy in the form of competency training in the generalist healthcare setting does not exist.

## Additional files

Additional file 1:
**Table S1.** Extended version of Colaizzi’s phenomenological method. Additional file 2:
**Table S2.** Symbolic representation of phenomenon.

## Abbreviations

AIDS: acquired immunodeficiency syndrome
HIV: human immunodeficiency virus
IV: intravenous
SMI: serious mental illness
WHO: World Health Organisation
