# Supplementary material for: The phenomenon of co-morbid physical and mental illness in acute medical care: the lived experience of Australian health professionals
Source: BMC Res Notes. 2015 Jul 7;8:295. doi: 10.1186/s13104-015-1264-z (PMC4494698; doi:10.1186/s13104-015-1264-z)
Supplement: Additional file 1: — Table S1. Extended version of Colaizzi’s phenomenological method. [file 13104_2015_1264_MOESM1_ESM.docx]

Table 1. Extended version of Colaizzi’s Phenomenological method

| Step | Description |
| --- | --- |
|  | Interview transcripts read to attain a feeling for them |
|  | Significant statements are extracted [statements that directly relate to the phenomenon under investigation and form the basic unit of analysis] |
|  | Formulated meanings are attached to each statement [researcher attempts to identify meaning to each statement] |
|  | The formulated meanings are grouped into theme clusters |
|  | An exhaustive description of the experience is developed [comprehensive description of the experience as described by the health professionals] |
|  | Added Step – the researcher interprets the symbolic representation of the lived experience presented in the interview |
|  | Identification of the fundamental structure of the phenomenon |
|  | Return to participants for validation and any new data revealed at this time is included to the final data collection |
