# Supplementary material for: The phenomenon of co-morbid physical and mental illness in acute medical care: the lived experience of Australian health professionals
Source: BMC Res Notes. 2015 Jul 7;8:295. doi: 10.1186/s13104-015-1264-z (PMC4494698; doi:10.1186/s13104-015-1264-z)
Supplement: Additional file 2: — Table S2. Symbolic representation of phenomenon. [file 13104_2015_1264_MOESM2_ESM.docx]

Table 2. Symbolic representation of phenomenon

| **Participant** | **Symbolic Representation provided by participant** | **Researcher’s interpretation** |
| --- | --- | --- |
| 1 | *For me personally eye opening, like I said never in my dreams had I experienced so many or thought that so many people would come through a department and never had I thought that the community was so mentally sick, so I would have to say eye opening and at times over whelming... just those two.* | Lacking the understanding and knowledge about mental illness makes one feel unprepared to deal with mental illness and this can be overwhelming. |
| 2 | *A roller coaster - you are going up and down, up and down, you may be making breakthroughs with that patient or they could be going up as in they are acutely aggressive, like they need medication and are so escalating as they are getting really aggressive they need to be restrained and then they need to be medically sedated and I guess the down could be the calming down they are breathing fine, the parents in there consoling them or they could just be sedated. Yes up and down, so the sedation wears off and they decide to come around again and decide whether they are going to be good or bad. You know that feeling in your stomach…when it kind of drops when you realize they are going to be difficult, they are going to try to hit someone or oh god when they are saying rude things to you, you just get that pit in your stomach.* | The care of patients with a mental illness evokes a sense of the unknown evoking apprehension. |
| 3 | *Buying a mystery flight. You can find out where you are going and can be disappointed because you have heard things about it that you don’t like or you can go in and say ‘ well I am on a holiday and just enjoy it...’ Things can happen but you just go with it. Similar to when at work; you go to work generally with a good feeling about your shift, you get given this patient and really you know nothing about this patient, you can be told things but you just have to go along and make the best of it. It can be unpredictable but generally it is your attitude towards it.* | The experience of caring for a patient with a co-morbid physical and mental illness is determined by the attitude towards mental illness and by seeing the patient for who they are beyond their diagnosis. |
| 4 | *It is just like a fishing net with big holes, where they are getting the care they need then just slipping through it. I feel it’s like a sieve, people get drained through, and they just don’t respond well when you are trying to help them. The gaps in their care make it very difficult to care for them. Patients can be very defensive from the get go because of their past experience. Some patients don’t want help, not ready to get help. Our hospital system is not geared for mental health follow up.* | Gaps in a health professional’s knowledge and the healthcare system that is not equipped to adequately care for patients with a co-morbid physical and mental illness makes the experience challenging for both the health professional and the patient. |
| 5 | *I guess a roller coaster ... in that it represents that emergency department (ED) situation accurately and also represents the dramatic difference between experiences in the ED and the experiences on a gen-med ward. In particular when you see a hostile situation and subsequent experiences after you do a psychiatric placement and realise that patients with mental illness are not that different from a normal patient, so a roller coaster.* | Mental illness can seem scary at first evoking feelings of apprehension and fear. Once you have experienced caring for patients who experience mental illness care is normalised as fear and apprehension decrease. |
| 6 | *You really feel like it is a vicious cycle, you try to help the patient and then it just seems to come back to bite you! Really it felt like a scary ride…a roller coaster going up down and you don’t know when you are going to freak out…but once the patient has settled and then it was like that finally the roller coaster has stopped…well for me anyway for the patient it may have been a whole other experience.* | Providing care for someone who experiences mental illness causes tension and uncertainty. Once things settle down those feelings subside for the health professional, however the experience may be different for the patient. |
